# Adult Perceptions of Healthy Pregnancy: A Focus-Group Study

**DOI:** 10.3390/ijerph17072460

**Published:** 2020-04-03

**Authors:** Hae Won Kim, Duck Hee Kim, Hyang Yuol Lee, Young Jin Lee, Hye Young Ahn

**Affiliations:** 1 Seoul National University, College of Nursing, Research Institute of Nursing Science, Seoul 03080, Korea; haewon@snu.ac.kr; 2 College of Nursing, Woosuk University, Jeollabuk-do 55338, Korea; dhkim0@woosuk.ac.kr; 3 College of Nursing, The Catholic University of Korea, Seoul 06591, Korea; 4 College of Nursing, Seoul National University, Seoul 03080, Korea; hygiene2@snu.ac.kr; 5 College of Nursing, Eulji University, Daejeon 34824, Korea; ahanaya@eulji.ac.kr

**Keywords:** pregnancy, preconception care, social perception, focus groups

## Abstract

The fastest aging society with the lowest fertility rate can be buffered by support for healthy pregnancies using sociocultural approaches. We aimed to address adult perceptions of a healthy pregnancy and explored their needs and concerns about childbirth across the lifespan. We conducted a qualitative study using content analysis to investigate general perceptions of a healthy pregnancy after focus-group interviews with adult men and women. We interviewed 60 participants in nine group sessions of 5 to 8 people per group. Three major themes emerged that affect healthy pregnancies: Taking responsibility for a prepared pregnancy, factors that interfere with a healthy pregnancy, and improving strategies for a healthy pregnancy. For the first theme, the two main concerns were financial and parenthood preparation. Factors interfering with a healthy pregnancy had direct and indirect causes, considering personal, social, and cultural changes. Strategies for a healthy pregnancy included family and workplace support, systematic education, and governmental support for financial preparation and health screening. Participants averred that various kinds of support (financial, healthcare, and career) are needed for a healthy pregnancy and childbirth. This public awareness could promote better decisions toward healthy pregnancy with more sociocultural approaches in the various settings of home, school, and the workplace.

## 1. Introduction

Koreans have experienced a steady increase in the rates of high-risk pregnancies with maternal age over 35 growing from 14.3% in 2008 to 31.8% in 2018 [1]. The total fertility rate in Korea (1.05) was the lowest in 2017 of developed countries [2] and fell to 0.98 in 2018 [3]. Mean mother age at childbirth was also the oldest in 2017 at 32.6 years old of the 34 countries that reported the measure [2]. Due to having the lowest birth rates, Korea has initiated public-policy options to encourage childbirth nationally and to face demographic changes as the fastest aging country in the world [4]. To facilitate a pregnancy, many healthcare professionals have focused on preconception- and interconception-care interventions, reducing high-risk factors and unmet needs from a healthcare providers’ perspective [5]. The timely implementation of a comprehensive program of preconception care and increasing awareness and knowledge of care from care providers and future parents became imperative [6]. However, no study investigated perceptions of healthy pregnancy with the general public as a target population. It is critical to know why people delay and do not plan for childbirth [7]. Before its implementation, understanding general perceptions of a healthy pregnancy from the public and listening to various families’ perspectives from different groups could provide more concrete direction as to how to facilitate healthy pregnancies in the community using a cultural and life-span approach. Korean society can better understand the decreasing rate of childbirth and increased high-risk pregnancy rates by understanding problems from a sociocultural and behavioral perspective. Therefore, for this article, we explored general perceptions of the public about healthy pregnancy and general concerns or unmet needs during pregnancy events across the life span.

The concept of preconception care emphasizes planning or preparation before childbirth to achieve a healthy pregnancy [8]. Preconception health refers to health across the entire lifespan of any man or woman prior to a potential pregnancy. If behavioral changes occur well in advance of pregnancy, through effective interventions, any adverse effects on the child can be prevented [9]. Although the Korean government does not provide official guidelines for preconception care and primary prevention of high-risk pregnancy across the lifespan, community-based maternal support in local healthcare institutions such as community-based public health centers provide preventive care for current or future pregnant women in various ways, such as providing folic acid and iron supplements [10]. To understand the macrophenomenon of gender, family, and generations to prevent high-risk pregnancy, it is critical to discern actual or perceived problems regarding healthy pregnancy from the general public. Such knowledge will provide a broader understanding of the issues of healthy pregnancy so a life-span approach can change people’s informed decisions about their life plan. With stronger sociocultural and behavioral changes occurring during pregnancy, rather than medical treatment and medication support, people may gain healthier natural pregnancies.

To better understand preconception care and healthy pregnancies, we retrieved a comprehensive theoretical framework from previous studies. The effectiveness of general preconception care could be delineated with Anderson’s model of healthcare using the three concepts of environment, targeted population, and outcomes [11]. In the framework for the Healthy Pregnancy 4 All model, developed to understand the use of preconception care in The Netherlands, preconception-care consultations and recruitment strategies are included in a local community’s internal environments, and many care organizations and networks provide other kinds of preconception care in external environments. Then, interactions are followed by predisposing characteristics, enabling resources, and needs within the targeted populations. The primary outcome of reducing preconception risk aims is to achieve thorough preconception care; the second outcome is a behavioral change in preconception risk behaviors. Multiple constructs could explain preconception stress and resiliency during childbirth that consider mothers’ and fathers’ resilience and social support in neighborhood and community contexts and the outer surrounds of healthcare, education, work, recreation, and spiritual resources [12]. Four public health models of primary care, hospital-based and interconception care, community-based care, and specific preconception-care clinics classify preconception care [13]. Pregnancy outcomes could vary by multiple determinants of integrated perinatal health: social, psychological, behavioral, environmental, and biological [14].

Although pregnancy is not only a personal decision but also a family and sociocultural one, huge differences may emerge in maternal experiences regarding pregnancy and childbirth. It would be meaningful to know how men prepare for a healthy pregnancy. As most women who prepare for childbirth expect substantial contributions to the process from the family [15], the responsibility of family members as supporters should be explored. Thus, it is worthwhile to explore the perceptions of family members on healthy pregnancies and childbirth processes.

We explored perceptions of healthy pregnancy using qualitative focus-group interviews to discern a comprehensive understanding from the general public. This study inquired about general perceptions of healthy pregnancies across the life span from men and women, unmarried and married. We questioned how they think about family and societal responsibilities in relation to healthy pregnancies.

## 2. Materials and Methods 

### 2.1. Study Design

This study entailed qualitative descriptive research with focus group interviews to gain a more comprehensive understanding of the perceptions of healthy pregnancies, revealing opinions and experiences of participants.

### 2.2. Study Participants

To facilitate active discussion among participants, we used to a purposive-sampling method to recruit participants who had similar characteristics and experiences. We recruited study participants aligned with characteristics of age, marital status, and childbirth experience: university students, unmarried young adults, married young adults, and married middle-aged adults (see Table 1). We formed groups of participants with similar characteristics to facilitate the discussion. To achieve theoretical saturation of collected qualitative data, participating numbers in focus groups should range from 3 to 12. Also, 7 to 10 participants per group is appropriate [16]. We conducted a total of 9 focus groups (A–I). To address the ongoing debate on the usefulness of mixed or homogenous groups, we formed seven mixed-gender groups (A–G) and two same-gender groups (H,I). Each focus group had 5 to 8 participants. We held 10 interview sessions, described in Table 1. Focus-group sessions explored participants’ experiences and opinions regarding healthy and high-risk pregnancy.

### 2.3. Data Collection

The first author (H.W.K.), with a background in women’s health and experience conducting qualitative research, moderated each focus-group session. The moderator worked to limit bias or assumptions about the study topic and had no relationships with participants prior to the study. A doctoral student (Y.J.L.), with training in qualitative research, assisted during the sessions. The assistant took notes and recorded audio. Eight (A–H) of nine groups were interviewed once, and one group (I) took part in two interviews (see Table 1). Sessions took place in a university conference room, participants’ offices, and participants’ churches to help them feel comfortable. The average time per session was 82 min, with a range of 63 to 110 min. All sessions took place July to September 2018. Participants responded to the following two open-ended questions.

- What does healthy pregnancy and childbirth mean to you and why does high-risk pregnancy occur?
- What do you think that are the roles of men and women, families, government, and society in preventing high-risk pregnancy?

All sessions were recorded using two separate digital recorders, with participants’ permission. At the end of each session, the moderator assessed each participant’s contribution and summarized the discussion, ensuring participants agreed with the summary. A research assistant transcribed the audio recording of each session to produce a verbatim report after listening to the recorded content repeatedly. Participants received financial rewards for transportation and their time investment (30,000 Korean won per person).

### 2.4. Ethical Considerations

The Institutional Review Board of Seoul National University approved this study (IRB No.1805/001-009), carried out in accordance with the Declaration of Helsinki. Participants received an explanation of the aims of the study and a guarantee of anonymity and confidentiality. After providing written consent, participants were enrolled in the study. We assured participants they could withdraw from the study at any time without explanation. A research assistant stored the transcribed data in a locked filing cabinet and on a password-protected computer.

### 2.5. Data Analysis

We analyzed data using the inductive content analysis methodology [17]. Two researchers (H.W.K. and D.H.K.) repeatedly read the transcribed contents line by line and derived meaningful concepts from the statements. They coded and named important concepts in the derived sentences and created and abstracted subcategories by categorizing content with similar codes. Finally, researchers grouped the abstracted categories with similar content to further abstract them to derive higher categories. To progressively refine the overall content, we iteratively matched the analyzed concepts with statements from the verbatim report. We analyzed qualitative data using NVivo 12.0 (QSR International, Victoria, Australia).

### 2.6. Study Rigor

To assure the quality of the study, we carefully considered internal validity, external validity, reliability, and objectivity of this qualitative research. For internal validity, one researcher led focus-group interviews and one research assistant participated in all sessions by following the group interview guide for every session. For external validity, we carefully assigned study participants to groups depending on their characteristics and designed focus groups systematically with the same or different characteristics to make them representative of the general target populations. Reliability of data was assured in that the research assistant transcribed all recordings, and the research team confirmed the verbatim report before data coding. For objectivity, one expert member (D.H.K.) of the research team who has participated in several qualitative studies but did not participate in the focus-group interviews coded and analyzed the data. The principal investigator (H.W.K.), who has participated in many qualitative studies, designed this study, guided interviewers, coded and analyzed data with D.H.K., and drafted a theoretical framework. The entire team discussed themes, categories, and subcategories describing general perceptions and concerns about a healthy pregnancy.

## 3. Results

Sixty participants took part in the study (34 women), and the average age was 35.6 years. The average age of 24 married participants who had at least one child at the last birth was 35.5 years. Table 2 presents participants’ characteristics.

### 3.1. Healthy Pregnancy—Responsibility Requires Preparation

For a healthy pregnancy to take place, all participants said they needed to be prepared in the following ways.

#### 3.1.1. Financial Preparation

Participants stated they had to be economically prepared to take care of their children so they could plan a pregnancy prior to give a birth. In Korean society, parents’ economic power must precede support for their children’s education. Without such preparation, it seemed irresponsible to have children.

- The biggest issue, people say, is a financial thing. In Korea, it is too expensive to educate children.… I think it is the first reason in our society why it is so easy to hear that people talk about giving birth with no responsibility. (Focus Group[FG]4, unmarried Woman[W]6)
- I think that the husband’s economic capacity is important because the process throughout the pregnancy and childbirth actually includes an economic piece. As was already mentioned, our psychological comfort is somewhat affected by economic support. If I get pregnant, I need to rest from my work in some ways, so I think the husband’s economic capacity may be important. (FG3, unmarried Man[M]2)
- Most couples get married but decide not to have a baby. Too much money is needed. All the reasons have to do with money. The biggest reason for not having a second baby is actually an economic reason (FGI9, married W3).

#### 3.1.2. Preparation for Parenthood

Most participants expressed that parents should be able to have children after planning, through sufficient dialogue with their spouse and deep consideration of their roles as a parent.

- Prior to pregnancy as well as birth, I think pregnancy should have planned ahead. I have seen a lot of cases when people got pregnant and then got married. I think that pregnancy should be planned and prepared thoroughly, medically and socially. (FG1, unmarried M6)
- Prepare for my health: I was talking about some medications before. Our pastor said that people should not be married without any preparation in how to be a father and how to take care of a child and must prepare for marriage, for example, by taking a course in marriage preparatory school before getting married and pregnant. (FG5, married W4)

1. Preparation of the physical body: Exercise and healthy eating habits. Many participants stated they should prepare their bodies to become pregnant, regardless of gender. They advocated habits of good body management, especially exercise and eating habits.

- I think men should also do health checks. In fact, men who are in their 40s have to be examined and some issues cannot be fixed at that time. So, it is better to begin and continue to get health checkups and exercise earlier while young. I actually exercise regularly. (FG3, unmarried M3)
- I think I should keep managing my body early before marriage. Because I could get pregnant right after I got married. But, if I were pregnant and my body condition were not ready for a child, then I think it is irresponsible to be a parent. (FG4, unmarried W3)
- I think I should improve my eating habits a bit. I also eat too much fast food and too much instant food, so I think I should improve that part. And, I’ve been hearing a lot about environmental hormones since I was a kid. I think I should pay more attention to that kind of thing. (FG3, unmarried M2)

2. Preparation of parental mind: To become a parent through a healthy pregnancy, the couple should have a plan for pregnancy and think about what to do and how to prepare for the child to be born.

- Continuing my job after childbirth is a big issue. It could be very stressful, and it is also a very big issue for the baby and mother’s health. Regarding this, I think the mother should communicate enough with her husband and make a mutual plan, so that potential risks from a pregnancy can be diminished, so we can do well later. (FG8, unmarried M2)
- I have thought a lot about mentality when I thought of my pregnancy. Living with a child is not physically easy. Changes in physical health also change perceptions. I want to give birth to three children, but my body may not be able to handle it. If so, I would feel stressed and depressed about that and also pressure my children. Then, I should not think of giving birth to too many children thoughtlessly. (FG4, unmarried W5)

3. Change individual perceptions about pregnancy and childbirth: In their 20s and 30s, many unmarried participants thought pregnancy and childbirth are “difficult” and “painful.” They expressed complex and diverse ideas, feeling a heavy sense of responsibility. These thoughts about pregnancy and childbirth dominated the younger generation’s conceptions of family, preventing them from choosing to have children. They prioritized their own happiness. Participants thought it necessary to change individual perceptions for more people to give birth.

- I have not thought of the idea of pregnancy and childbirth as good since I was a child. Of course, I would like to raise a child who resembles me, but I am afraid that the process is so painful. I have no confidence in the process of enduring all that pain. I do not seem to be well aware of the way I see things often. (FG1, unmarried F1)
- In fact, the value of home, marriage, pregnancy, and childbirth does not really touch me well. Why should we marry? I do not see why I should pack up my family and why I should be pregnant. I can only say that I am indifferent to such things, and there are more people like me. It seems to be contributing to a low birth rate. I still have not changed my mind. I think the value of pregnancy and these things is not acceptable. (FG1, unmarried M3)
- I think that the value of connecting a household with the family was very important in my mother and father’s generation, but it seems that the value of my family is really blurred and the value of happiness is more important in my generation. Mom and Dad say to me, “If you want to give birth, do it; if you do not want to give birth, do not give birth.” But, I do not know if I would hear that from my grandfather. (FG1, unmarried M6)

### 3.2. What Are the Barriers to Healthy Pregnancy?

Participants did not know much about high-risk pregnancies. Particularly, unmarried young participants were unfamiliar with the term and sometimes referred to an ectopic pregnancy. They thought the term “pregnancy addiction” referred to frequent pregnancies. Some participants knew indirectly about high-risk pregnancies through family or friends or through the media. They said a high-risk pregnancy meant the mother was physically ill or was ill prepared for pregnancy. Participants raised the notion that Korean women are different from the past and pointed out the inadequacy of education on high-risk pregnancies.

#### 3.2.1. Direct Causes Related to High-Risk Pregnancy

1. Old age: Most participants referred to the age of the future mother as a factor in high-risk pregnancy. Participants considered that various personal and social factors about marriage and childbirth delayed marriage and, as an outcome, caused high-risk pregnancies and late childbirth.

- In my opinion, the age to get a job is delayed; then, the age to marry is also delayed, and the age to prepare for a house is delayed. [High-risk pregnancy] seems to be naturally occurring. (FG8, unmarried M5)
- As far as I know, a mother gets pregnant with a physical disease or is not ready for pregnancy. I heard that the mother’s older age is going to be more than mid-30s for high-risk pregnancy. (FG3, unmarried W5)
- My mother always says, “so you should get married as soon as possible, and when you get much older, then you may not be able to even have a baby.” (FGI2, unmarried F2)

2. Diseases and Stress: Participants said that various stresses, such as work and family conflicts, may cause high-risk pregnancies as well as underlying diseases.

- I also remember that pregnant women with diabetes and hypertension and so on is called a high-risk pregnancy. (FG3, unmarried F6)
- At first, I had an irregular menstrual cycle due to polycystic ovary syndrome. I have not been pregnant for a year. Then, I went to the hospital and was prescribed medication. (FGI6, unmarried W2)
- I felt stress as I got older and stressed more when things come. I felt like I had some worse results. (FG8, unmarried M5)

#### 3.2.2. Indirect Causes Related to High-Risk Pregnancy

1. Changes in women’s values and perceptions: Young women in their 20s and 30s have quite different perceptions about pregnancy and childbirth from their mothers. Young participants said they would choose marriage or pregnancy as a choice rather than an obligation or responsibility and with a firm preference for an independent life and thorough preparation. Some participants perceived their life changes as becoming irreversible throughout a pregnancy.

- I am 42 and working. My colleagues around me are 38, 36, and 34 who are all unmarried. When I ask why not, they said, “I have a boyfriend, but I do not feel the need for marriage.” I think they are able to be responsible for themselves, so they seem to find others similar to themselves. And, there is something annoying about a child. ”Why should I do such a thing because of the baby?” (FG7, married W8)
- I tend to think that pregnancy and childbirth are somewhat irreversible because, before and after the process of pregnancy and childbirth, there are many changes, whether physical or mental, personal, or social, anyway. It may be a little dangerous, a process that is destroying a process in my life, or a process of living just by taking a physical risk. When I think about it a little bit, I think it is impossible to recover. The feeling of being in an unrecoverable situation rather than a transition period, I think that’s a negative thought. (FG1, married W5)

However, women of the parents’ generation thought marriage should be a process of life and parents should be devoted to their children, recognizing pregnancy as a noble aspect.

- I married when my husband was in the military. At that time, I was not mature, so I thought that if I really had love, I would live, and I thought it was all done with just getting married. I got married without thinking about the culture of my husband’s family. (FG5, married W2)
- Young couples, I wish they knew that the process of birth is worthwhile. (FG7, married W2)
- We have to work hard in our generation to be dedicated to our children, but our kids are not like our parents. Our kids say, “I’m free. Unlike my mother and father, I’m making money to go travel and I’m not going to be interrupted.” I would like them to change the concepts of marriage. If the concept of marriage changes, the rest of the issues will be solved. (FG5, married W1)

2. Lack of (poor) school and home education about sex: Sex education in school is quite basic and biologically oriented, so knowledge related to the process of pregnancy and childbirth is insufficient among adults.

- I do not know exactly what we are talking about, even though we are in the mid-20s, as to what is high-risk pregnancy. It is a problem that should be treated in sex education. So, I thought that if I knew a little more, I could prepare and plan. (FG3, unmarried M2)
- If you look at young people or those people, you can have sex without thinking and get pregnant before you are ready. If so, a high-risk pregnancy or something like that might happen. I think men need to know more about high-risk pregnancies. I think it would be nice to know some more about if there are precaution strategies. I did not learn about high-risk pregnancy in sex education. (FG8, unmarried M4)

In Korea, many schools provide very basic sex education. Talking about sexuality related to pregnancy and childbirth is not easy for parents to communicate at home or through children’s education.

- Sex education, it is important in the school, but in the family, I think I should learn it from childhood. I have heard something in foreign countries that they are saying these things are fairy tales from childhood, but it is not so in Korea. We think it is a shame. (FG3, unmarried W4)
- My husband did not teach my children regarding masturbation during our boy’s puberty. We have to do that (education), but we could not do that because it was awkward. (FG5, married W1)

### 3.3. Improving Strategies for Healthy Pregnancy

For healthy pregnancies, participants suggested the sociocultural environment and family support improved, as did the need for education and substantial support in pregnancy, childbirth, and prevention of high-risk pregnancy.

#### 3.3.1. Family (Parental) Support

If parents’ interest in their children’s marriage and pregnancy is quite intense, the unmarried couple may feel burdened by their parents’ desire to have grandchildren. Parents should let their children decide for themselves if they want to marry and have children. If parents can help their children in child raising, children will consider giving birth at a younger age. Also, it may be easier for young women to keep their jobs.

- Nowadays, parents should not force their child to get pregnant. I think pregnancy should be planned under the couples’ own will. If someone around me presses me about why I don’t have a baby, then I may not be prepared. (FG8, unmarried M2)
- It seems to be good to reduce the burden of being attached to a certain sex (girl or boy) mentally. I do not think that many people can do that in a positive way, such as if I am in a position to be able to care for a child later by buying a house near my parents’ house. There may be a negative part, but a positive part is helpful for me. I think that’s part of the role of the family too. (FG8, unmarried M2)

#### 3.3.2. Support in the Workplace

For working women, discontinuing their career is considered a disadvantage for gaining promotions and maintaining a career. Therefore, they do not choose marriage, thereby delaying pregnancy, resulting in the serious problems of giving birth at an older age. Participants thought it necessary to support healthy pregnancy and childbirth at the social level.

- It is a same matter for a man or woman, more than anything else in pregnancy and childbirth in Korea. Is it a social restriction? I think I should be rewarded with a little more social benefit. When women give birth to a baby, women need to take maternity leave so they can give birth freely. I think my job should be guaranteed when I go on vacation. (FG8, unmarried M5)
- I have to be promoted, but it seems delayed because of childrearing. This kind of invisible discrimination exists from the time of planning a pregnancy to the childbirth, even though I do not tell anyone that I seem to be getting stressed. (FGI9, married W3)

#### 3.3.3. Building a Systematic Education System

Participants hoped educational programs would be organized systematically at the national, social, and institutional levels because knowledge of healthy pregnancies or preventing high-risk pregnancies is presently unavailable.

- I want to receive education like that about high-risk pregnancy. I think we need that too. So, we get to know about it and prepare for it so that we can make it. Then, if we eat appropriate food in a good environment, it will help us much more. We did not have such education or such a system at all. I’m just going to be a mom. (FG7, married W2)
- I think we should be educated by the nation. It would be awesome if we had something like pregnancy, childbirth, or high-risk pregnancy in our national education policy. (FG8, unmarried M2)
- I think it is necessary to start with things like pregnancy and the risk of giving birth, starting from adolescence and teaching it precisely. (FGI2, unmarried W1)

In addition, if educational information can be provided through various media, prevention and coping with high-risk pregnancies would be technically easier and more common.

- Nowadays, it is better to find a website such as a mom café. I would use it when I have a baby or later.… What is the role of a man and a woman in the case of high-risk pregnancy? If there are easier ways to find better information in apps or in other ways, I would choose those ways. (FG8, unmarried M2)

#### 3.3.4. Substantial Support for Pregnancy/Childbirth/Childrearing

Many participants were unaware of the extent to which the nation actually provided support for pregnancy, childbirth, and childrearing. However, commonly, participants raised the issue of national support, and some participants suggested it should be possible to support pregnancy-related health screening for women who signed up early.

- I do not know what kind of support is being given in the country right now. (FG3, unmarried W7)
- The first problem is policy as well, and there should be a lot of support for childbirth and childrearing. (FG7, married M6)
- I do not know where I am now. If we say a high-risk pregnant woman is older than 35 years old, supporting health checkups or only screening for pregnancy-related items into the 30s or late 20s, would that be helpful? (FG3, unmarried M2)

### 3.4. The Healthy Pregnancy Model 

Table 3 explains the contents of our study findings.

## 4. Discussion

This study provided new information, identifying general perceptions and socioculturally unmet needs toward healthy pregnancies. The Republic of Korea faces decreasing childbirth rates and increasing high-risk pregnancy rates. The first theme described a long waiting period to be sufficiently financially secure, prior to a healthy pregnancy, along with physical and psychological preparation for mature parenthood. Delaying pregnancy and preparing financially to raise a child means that Koreans delay personal health preparations to have a healthy pregnancy. Although pregnancy and childbirth are personal decisions, the social and cultural environment impacts the life process. Study participants expressed that economic support seems to be a prerequisite for pregnancy, childbirth, and childrearing. Hence, the phenomenon of low fertility in Korean society reflects an economic decision of not giving birth. Participants had negative perceptions about pregnancy, perceiving personal changes due to pregnancy and childbirth as unfavorable, thereby causing them to delay marriage and pregnancy. Such delay becomes a reasonable decision to protect their personal happiness and to improve their quality of life without the children. It is necessary to understand individual perceptions that pregnancy is not connected to personal happiness. People decide not to give a birth because they think a child will not help them live a personally happy life. 

For the second theme of factors interfering with healthy pregnancy, participants recognized the necessity for preconception care. They understood that health meant no alcohol, no smoking, and taking nutrients like folic acid. They were also aware that chronic-disease patients should take medications after consulting with a doctor. Furthermore, future mothers aimed to decrease work-related tasks before pregnancy because the perception was that personal stress control is the main component in maintaining a healthy pregnancy.

Although participants indicated that a mother’s older age was the immediate cause of high-risk pregnancy, most perceived that maternal diseases and stress could be prevented and managed earlier. Previous researchers found that maternal age is not a factor in high-risk pregnancy and that self-management during pregnancy can increase older pregnant women’s nutritional status and fetal health [18]. Another study using national childbirth data from 2003 to 2009 noted that, for older pregnant women, age itself could be a risk factor for low birth-weight infants and premature birth, whereas good preconception care can make normal childbirth possible; thus, preconception care is more important than the mother’s age [19]. In our findings, perceived risks can be reduced through continuous efforts toward preconception care and stress management for healthy pregnancies.

Lack of education seems to be an indirect cause of high-risk pregnancy in that current sex education in school is ineffective and perfunctory. The current content of public education about sex should be modified to include healthy pregnancy. Participants also suggested that schools should provide customized sex education on healthy pregnancy and potential parents should self-educate at home.

The third category of strategies for healthy pregnancy includes social change, allowing one’s family to provide support for childrearing and career support for pregnant women through systematic education initiated by the government, along with the provision of actual financial support and free health screenings. Most workplaces do not allow people to take personal time for education, so people prefer online media to offline education. Information can be shared easily through the community, with media offering reliable education [20]. Therefore, the Korean government could provide (a) realistic preconception-care guidelines for a broad range of potential parent-to-be populations, (b) online information support, (c) increased public-relations support for healthy pregnancy, (d) preconception-care education through open communication by specialists in healthcare organizations and community health centers, and (e) 24-hour counseling hotlines. Through these mechanisms, the general public can be educated and future parents can achieve healthy pregnancies through community-based services.

This study has strengths and limitations. Many recent studies excluded male participants [9]. We included men and women to capture how people of different genders express concerns. Participants included married and unmarried men and women with no children, one child, or many children. This breadth might be a limitation or the strength of this study, providing an innovative approach and possible direction for future research.

## 5. Conclusions

Study participants expressed their perceptions and attitudes about healthy pregnancy and risk factors and described strategies to support healthy pregnancy and childbirth. A social and cultural support system in the family and at the workplace could facilitate healthy pregnancy. A standardized practice guideline for preconception care is needed to encourage home- and community-based education in local provinces. Public education programs to prepare future mothers and fathers could provide valuable information, allowing better decisions. The community, home, and workplace could provide various kinds of resources for pregnancy and childrearing. We comprehensively explored healthy pregnancy with members of the general population. Community-based interventions will help in establishing a culture for healthy pregnancy.

## Figures and Tables

**Table 1:** The compositions of participants in groups and interview time of each session.

Focus Group	No. of Participants	Gender		Age			Marital Status	Duration of Interview (min)	Interview Time
		Male	Female	Range	M	SD		(M ± SD)	
A	7	4	5	21–24	22.57	0.98	Unmarried	77	Evening
B	5	2	3	25–32	28.60	3.29	Unmarried	72	Evening
C	7	3	4	20–25	23.29	2.06	Unmarried	73	Evening
D	6	2	4	26–35	30.83	3.43	Mixed	93	Afternoon
E	8	3	5	50–62	57.25	3.81	Married	82	Afternoon
F	7	4	5	31–39	34.43	2.76	Married	108	Evening
G	8	3	5	42–52	46.38	3.66	Married	110	Afternoon
H	5	5		32–34	32.80	0.84	Unmarried	72	Evening
I-1	6	-	6	33–37	34.33	1.75	Married	63	Afternoon
I-2	7	-	7	33–44	35.71	3.99	Married	68	Afternoon
Total	60	25	35	20–62	36.26	11.67		81.80 ± 16.48	

M: Mean; SD: Standard deviation.

**Table 2:** General characteristics of 60 participants.

Characteristics	Total				Male (*n* = 26)				Female (*n* = 34)			
	*N*	%	*M*	*SD*	*n*	%	*M*	*SD*	*n*	%	*M*	*SD*
Age			35.55	11.52			34.62	11.48			36.26	11.67
20–29	19	31.7			8	30.8			11	32.4		
30–39	24	40.0			12	46.2			12	35.3		
40+	17	28.3			6	23.1			11	32.4		
Marital status												
Single	28	46.7			15	57.7			13	38.2		
Married	32	56.7			11	42.3			21	61.8		
Experience of pregnancy ^1^												
Yes	24	40.0			7	26.9			17	50.0		
No	36	60.0			19	73.1			17	50.0		
Experience of birth ^1^												
Yes	21	35.0			6	23.1			15	44.1		
No	39	65.0			20	76.9			19	55.9		
Number of children (*n* = 24)			2.61	1.16			3.33	0.82			2.33	1.18
Age at last pregnancy or birth (*n* = 24)			35.5	4.75			37.00	3.42			34.88	5.16
Education level												
High school	14	23.3			7	26.9			7	20.6		
College	35	58.3			14	53.8			21	6.18		
Graduate school	11	18.3			5	19.2			6	17.6		
Religion												
Yes	44	73.3			17				27	79.4		
No	16	26.7			9				7	20.6		

^1^ If the respondent was a man, he answered about his wife’s pregnancy and birth experience.

**Table 3:** Perception of healthy pregnancy.

Core theme	3 Categories	8 SubCategories	12 Codes	18 Initial Codes
Healthy pregnancy influencer	*Theme 1:* Prepared pregnancy that can take responsibility	Financial preparation	Economic power	Child support expense
		Preparation for parenthood	Preparation of the body	Exercise
				Healthy eating habits
			Preparation of the mind	Planned pregnancy
				Stress management
			Perception changes about one’s parental role in members of the younger generation	Need of alternatives for negative perception about pregnancy and childbirth
	*Theme 2:* Factors that interfere with healthy pregnancy	Direct causes	Old age	Advanced maternal age
			Diseases and stress	Chronic disease
				Stress
		Indirect causes	Changes in modern women’s values	Focus on self-centered life
			Lack of education at school and home	School education
				Home education
	*Theme 3:* Improving strategies for healthy pregnancy	Support in the family	Parent’s assist, help for childrearing	Parent’s support for child-caring
		Support in the workplace	Ensure maternity rights at work	Ensuring career of working women, maternity leave
		Support of systematic education	Building educationalsystem	Preparing an education program
				Providing education through various media
		Support of the government	Substantial support for pregnancy/childbirth/childrearing	Financial support
				Health screening support
